# Real-World Outcomes of Palbociclib with Endocrine Therapy in HR+/HER2− Metastatic Breast Cancer: A Retrospective Study from Saudi Arabia

**DOI:** 10.3390/cancers18081270

**Published:** 2026-04-16

**Authors:** Abdalrhman H. Alanizi, Sarah N. Al-Shaiban, Reema Alotaibi, Reem Qubaiban, Esra’a Khader, Ahmed S. Alanazi, Hatoon Bakhribah, Nawal Alsubaie, Amani S. Alrossies, Sireen Abdul Rahim Shilbayeh, Ammena Y. Binsaleh

**Affiliations:** 1 Pharmaceutical Care Services, King Abdullah bin Abdulaziz University Hospital, Riyadh 11671, Saudi Arabia; 2 Department of Pharmacy Practice, College of Pharmacy, Princess Nourah bint Abdulrahman University, P.O. Box 84428, Riyadh 11671, Saudi Arabia; 3 Department of Pharmacy, Al Kharj Military Industries Corporation Hospital, Al-Kharj 16417, Saudi Arabia; 4 National Cancer Center, Riyadh 13315, Saudi Arabia; 5 College of Pharmacy, Department of Pharmacy Practice and Science, University of Iowa, Iowa City, IA 52242, USA; 6 Clinical Pharmacy, Pharmacy Service Administration, King Fahad Medical City, Riyadh 11525, Saudi Arabia; 7 Comprehensive Cancer Center, King Fahad Medical City, Riyadh 11525, Saudi Arabia

**Keywords:** palbociclib, CDK 4/6 inhibitors, HR-positive, HER2-negative, metastatic breast cancer, real-world data, Saudi Arabia

## Abstract

**Background:** Hormone receptor-positive (HR+), Human Epidermal growth factor Receptor 2 (HER2-negative) metastatic breast cancer (MBC) represents a substantial proportion of breast cancer cases in Saudi Arabia. Despite the established efficacy of cyclin-dependent kinase 4 and 6 (CDK4/6) inhibitors, particularly Palbociclib, in randomized control trials, real-world data from local institutions in Saudi Arabia remain limited. **Objectives:** This study aimed to evaluate progression-free survival (PFS), overall survival (OS), and toxicity profile among HR+, HER2-negative MBC female patients treated with Palbociclib at King Fahad Medical City (KFMC). **Methods:** A retrospective study was conducted on female patients with HR+/HER2-negative MBC treated with oral palbociclib combined with endocrine therapy (ET) at KFMC between January 2021 and September 2024. Data were collected from electronic health records. Descriptive statistics were conducted using mean for continuous variables and frequency for categorical variables. Survival analyses were conducted using Cox regression, log-rank tests and Kaplan–Meier analysis. **Results:** A total of 169 female patients with HR+/HER2− MBC were included. In the first-line setting, the median PFS was 20.14 months (95% CI: 14.65–30.49), compared with 11.3 months (95% CI: 7.98–not estimable) in the second-line setting. For OS, the median OS values were 53.1 months (95% CI: 41.2–not estimable) in the first-line group and 23.7 months (95% CI: 18.5–not estimable) in the second-line group. Significant predictors of shorter PFS included age, Body Mass Index (BMI), type of ET, cancer type, line of therapy, family history of cancer, and history of VTE. Visceral metastasis (HR = 3.087; *p* = 0.0229) and ECOG performance status of 4 (HR = 13.86; *p* = 0.0156) were associated with significantly shorter OS. The most common hematological adverse events (AEs) were neutropenia (45.6%), followed by anemia (5.9%), leukopenia (5.3%), and back pain (5.3%). Most toxicities were managed with dose reduction, holding treatment, or supportive care. **Conclusions:** Palbociclib demonstrated favorable survival outcomes and a manageable safety profile, with neutropenia being the most common AE. This study provides region-specific real-world evidence supporting the use of Palbociclib in HR+/HER2− MBC. These findings align with global trial data and highlight the importance of individualized treatment in clinical practice.

## Simple Summary

Hormone receptor-positive, HER2-negative metastatic breast cancer is the most common form of advanced breast cancer. Palbociclib combined with hormone therapy has shown strong benefits in clinical trials, but local real-world data from Saudi Arabia are limited. This study evaluated the effectiveness and safety of palbociclib in 169 women treated at a large cancer center in Riyadh. The results showed that many patients achieved good disease control and meaningful survival outcomes. Side effects were generally manageable, with neutropenia being the most frequent, but serious complications were uncommon. These findings confirm that palbociclib is an effective and tolerable treatment option in routine clinical practice and provide important regional evidence to support treatment decisions for women with advanced breast cancer.

## 1. Introduction

Breast cancer (BC) is recognized by the World Health Organization (WHO) as the second most common cancer worldwide and the fourth leading cause of cancer-related mortality. In Saudi Arabia, BC is the first-ranked cancer among females with a significant increase in incidence over the years, accounting for 28.3% of all new cancer cases in 2022 [1].

Hormone receptor-positive (HR+) breast cancer represents 70% of the cases and has traditionally been treated with endocrine therapy (ET) like selective estrogen receptor modulator (SERM) and aromatase inhibitors (AIs) [2]. Metastatic breast cancer (MBC) and inoperable locally advanced breast cancer (LABC) are both types of advanced breast cancer (ABC) [3]. As MBC is generally not curable, the primary objective of care is to enhance both the length and the quality of the patient’s life [4].

In Saudi Arabia, more than half of breast cancer cases are identified late, compared with 20% in advanced countries. Consequently, this leads to higher mortality rates from breast cancer, diminished prospects for effective treatment, and increased healthcare costs [5].

The treatment of metastatic HR+/ Human Epidermal growth factor Receptor 2 Negative (HER2-negative) breast cancer is primarily based on ET [6]. Several agents, including selective SERMs and AIs, have been used as first-line options. In premenopausal patients, ovarian function suppression (OFS) is mandatory, so both pre- and postmenopausal women are now generally considered for AIs as first-line therapy in combination with OFS when needed [6,7]. Selective estrogen receptor degraders (SERDs), such as fulvestrant, are an option for both groups and can be used in either first- or second-line settings.

Targeted medications have been approved to treat HR+, HER2-negative MBC. The class of cyclin-dependent kinase 4 and 6 (CDK4/6) inhibitors has received a focus in treating MBC [8]. Palbociclib was the first CDK4/6 inhibitor approved in 2016 for the treatment of HR+, HER2-negative MBC in combination with AIs, based on the results of the PALOMA-1 trial [9,10]. Currently, three CDK4/6 inhibitors have been approved for clinical use: Palbociclib, Ribociclib, and Abemaciclib [3]. Palbociclib acts as a selective inhibitor of CDK4/6, which plays a crucial role in regulating the cell cycle [11].

According to the NCCN Clinical Practice Guidelines in Oncology: Breast Cancer [12], the recommended first-line treatment for HR+, HER2-negative\MBC is a CDK4/6 inhibitor combined with ET, for pre-/perimenopausal and postmenopausal. These agents have demonstrated significant improvements in progression-free survival (PFS), with an acceptable toxicity profile based on multiple clinical trials. However, ribociclib is the only CDK4/6 inhibitor that has both PFS and overall survival (OS) benefit and has favorable cost-effectiveness [3,13]. Similarly, the European Society for Medical Oncology (ESMO) guidelines recommend the use of a CDK4/6 inhibitor in combination with ET as the preferred first-line treatment for HR+/HER2− MBC [3]. Consistent with international recommendations, the National Cancer Center at the Saudi Health Council guidelines suggest that for postmenopausal women, the preferred first-line ET is an AI in combination with a CDK4/6 inhibitor [4].

PALOMA-2 validated the findings of PALOMA-1, showing that the combination of palbociclib and letrozole (AI) significantly improves PFS compared with the placebo-plus-letrozole regimen in HR+/HER2− ABC. The median PFS values were 24.8 months with palbociclib and 14.5 months with placebo. The primary endpoint of the PALOMA-2 trial was PFS, with OS being a secondary endpoint [14,15]. The trial was conducted across 17 countries in Europe, North America, and the Asia-Pacific region, with the majority of patients being recruited from the Russian Federation, the United States, Ukraine, Canada, and Belgium [14]. The OS results were announced in 2022 from the Phase 3 PALOMA-2 trial, which assessed the efficacy of palbociclib combined with letrozole versus placebo plus letrozole for first-line treatment in postmenopausal women with estrogen receptor-positive (ER+), HER2-negative MBC. With a median follow-up of 90 months, the palbociclib-plus-letrozole combination demonstrated a longer OS than placebo plus letrozole (53.9 vs. 51.2 months), though the difference was not statistically significant (HR = 0.956) [14,15].

The adverse event (AE) profile showed no variation across the trial reports, with the most frequently reported AEs being all-grade neutropenia (78.8–84.1%), leukopenia (39–60%), and fatigue (37.4–44.1%). Although neutropenia rates were high, febrile neutropenia remained uncommon, occurring in up to 2% of patients. Palbociclib plus fulvestrant maintained quality of life and significantly delayed progression in endocrine-resistant patients, as demonstrated in the PALOMA-3 trial [16].

In the Middle East, a retrospective real-world study conducted in Qatar by Al-Ziftawi et al. provided valuable regional evidence on the use of CDK4/6 inhibitors in the treatment of HR+/HER2-negative MBC [17]. The results showed a median PFS of 17.85 months in the palbociclib group. Similarly, median OS was 29.82 months for palbociclib. This study included highly diverse multinational populations, which may limit the generalizability of its findings to the Saudi population [17].

In Saudi Arabia, a retrospective study by Al-Foheidi et al. evaluated the clinical outcomes of palbociclib combined with ET among 97 patients who progressed on first-line therapy [18]. The study reported a median PFS of 16.3 months and a median OS of 28 months, supporting the efficacy of this regimen in real-world practice within the Saudi population [18]. While the study by Al-Foheidi et al. provided valuable insight into real-world outcomes of palbociclib in Saudi Arabia, it was limited by a small sample size, second-line setting, and lack of adjustment for key confounders.

The present study builds upon this evidence by including a larger sample, different settings of line of therapy, and more comprehensive statistical analyses, offering a stronger and more representative evaluation of palbociclib effectiveness and safety within the Saudi population, in alignment with regional and international findings [14,17].

Therefore, the objectives of this research study are to evaluate the effectiveness and safety of palbociclib in the treatment of HR+/HER2-negative MBC among female patients in Saudi Arabia. By analyzing real-world data, this study aims to assess PFS, OS, and treatment-related AEs within this population, providing insights into regional variations in treatment response and outcomes.

## 2. Materials and Methods

### 2.1. Study Setting

This retrospective observational study was conducted at KFMC, a tertiary care hospital in Riyadh, Saudi Arabia. The hospital provides specialized oncology services.

This study included all female patients diagnosed with HR+/HER2-negative MBC who initiated palbociclib between January 2021 and September 2024. The end of the follow-up period was January 2025, with a minimum follow-up period of 3 cycles. The date cut-off for all survival and safety analysis was defined as January 2025.

### 2.2. Study Population and Sample

We included female patients aged 18 years and older with a confirmed diagnosis of HR+, HER2-negative MBC who were prescribed Palbociclib in combination with ET, including AIs, SERMs, or SERDs, between 1 January 2021 and 31 December 2024.

To minimize selection bias, all consecutive eligible patients during the study period were retrospectively reviewed.

Patients were excluded if they were male, had been treated with another CDK4/6 inhibitor, had missing medical records, or did not complete at least one treatment cycle, which is defined as oral palbociclib once daily for 21 days followed by 7 days off [19].

**Figure 1:**
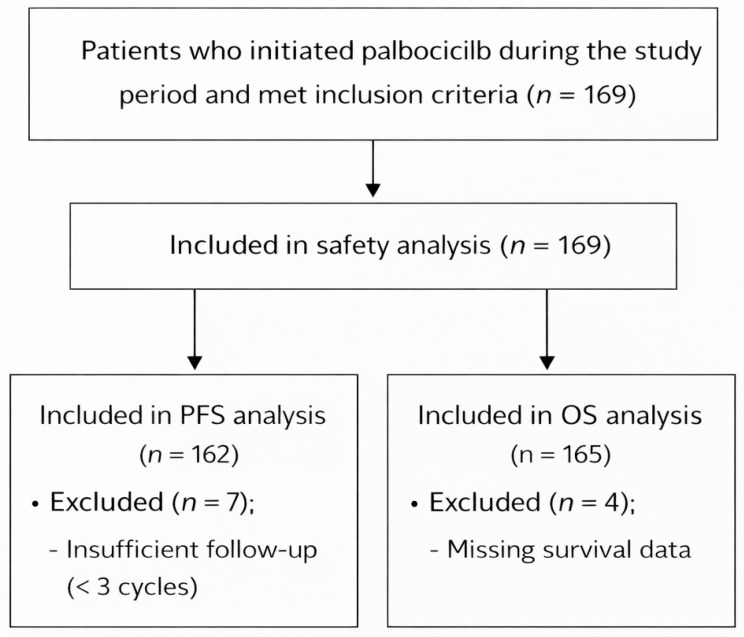
CONSORT-style flow diagram of patient inclusion and analysis. A total of 169 patients who initiated palbociclib during the study period met the inclusion criteria and were included in the study.

A total of 169 patients who initiated palbociclib therapy during the study period were included in the safety analysis, regardless of treatment duration, to ensure that early-onset adverse events were adequately captured. For effectiveness analyses, patients were included based on data availability for each outcome. A total of 162 patients were eligible for PFS analysis after excluding those with insufficient follow-up (<3 cycles), and 165 patients were included in the overall OS analysis after excluding those with missing survival data. The three-cycle threshold was selected to allow for a meaningful evaluation of treatment effectiveness and disease progression in a real-world setting [14]. Patient selection and inclusion are illustrated in Figure 1.

### 2.3. Outcome Measures

The primary outcome was PFS, defined as the time from the date of initiation of palbociclib therapy until disease progression or death by any cause, whichever came first. Disease progression was determined based on the treating physician’s assessment as documented in the patient’s electronic medical record (EMR), which included both clinical evaluation and radiological reports. Due to the retrospective nature of this study, standardized RECIST criteria were not uniformly applied. The secondary outcome includes OS, defined as the time from the date of initiation of palbociclib therapy until death [20,21]. The toxicity profile was evaluated based on severity and frequency of AEs using Common Terminology Criteria for Adverse Events (CTCAE) version 5.0. [22]. Additional outcomes included treatment discontinuation rates.

### 2.4. Data Collection and Handling

Data were collected from KFMC’s EPIC EMR system, including patient demographics, Eastern Cooperative Oncology Group (ECOG) performance status, metastatic disease locations, treatment history, palbociclib treatment duration, and reported AEs.

PFS and OS data were extracted based on physician-documented disease progression or death. All data were anonymized and coded for confidentiality prior to analysis, and no direct patient contact occurred.

Data preprocessing was performed prior to analysis, including verification of data completeness, consistency checks, and standardization of variable formats. Implausible or inconsistent entries were reviewed against the electronic medical record, where possible. Variables were coded appropriately prior to statistical analysis. Missing data in selected baseline variables (e.g., ECOG performance status and history of cancer) were handled by introducing a separate “not recorded” category to preserve sample size and reflect real-world data limitations. For PFS and OS outcomes, a complete-case analysis approach was applied.

### 2.5. Statistical Analysis

Descriptive statistics were summarized for patient characteristics and treatment patterns using means and standard deviations for continuous variables and frequencies with percentages for categorical variables.

Survival outcomes, PFS and OS were analyzed using Cox proportional regression models and Kaplan–Meier. Progression-free survival (PFS) time was calculated by taking the difference between the disease progression date and the palbociclib initiation date. However, if the patient did not experience disease progression, the follow-up time was set to be the difference between the end of the study date (31 December 2024) and the Palbociclib initiation date. Overall survival (OS) time was calculated by subtracting the Palbociclib initiation date from the death date. Log-rank tests and Cox proportional regression were used to explore factors associated with survival outcomes. Variables included in the multivariate Cox proportional model were selected based on clinical relevance and previously established prognostic factors in HR+/HER2− MBC, including age, BMI, ECOG performance status, and site of metastasis. Patients were censored if they did not experience the outcome of interest by the end of the study (31 December 2024). Models were adjusted for age, BMI, cancer type, endocrine therapy, family history of cancer, history of VRE, and line therapy.

Hazard ratios (HRs) with 95% confidence intervals (CIs) were reported. A *p*-value < 0.05 was considered statistically significant. Analyses were conducted using R Studio version 4.2.2.

### 2.6. Ethical Considerations

This retrospective real-world study was conducted following established ethical standards and was approved by the Institutional Review Board (IRB) of the Riyadh Second Health Cluster at King Fahad Medical City (IRB Log Number: 24-619)

Due to the retrospective nature of the study and the use of anonymized data, no direct patient contact or informed consent process was required.

## 3. Results

A total of 169 female patients with HR+/HER2-negative MBC were included in the analysis. All patients received palbociclib in combination with ET, including AIs, SERMs, or SERDs.

### 3.1. Characteristics of the Patients

The mean age was 58.4 ± 10.9 years, and the mean Body Mass Index (BMI) was 30.2 ± 7.9 kg/m^2^. ECOG scores of 1 (32.5%) and 2 (30.8%) were the most common, while 9.5% had a score of 4. Hypertension (49.7%) and diabetes (45.6%) were the most frequent comorbidities, followed by hyperlipidemia (22.5%). Other conditions were less common.

A history of prior malignancy was reported in 13% of the study population, and 15.4% had a family history of cancer. BRCA mutations were positive in 22.5% of patients. Most patients presented with de novo metastatic disease (71.6%). Palbociclib was used as first-line therapy in 82.2% and as second-line treatment in 17.8%. Postmenopausal women represented the majority (77.5%). Regarding metastatic distribution, 46.7% had both visceral and non-visceral disease. AIs were the most common endocrine therapy (65.1%). Full demographic and clinical details are shown in Table 1.

### 3.2. Progression-Free Survival

#### 3.2.1. First-Line Setting

**Figure 2:**
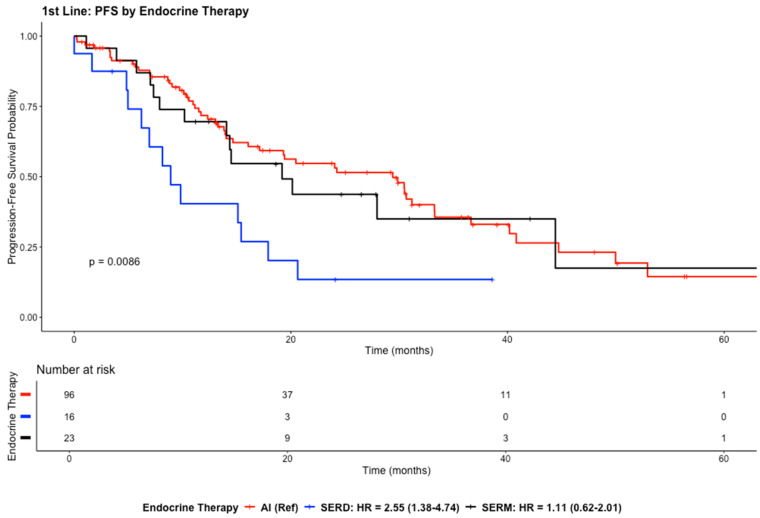
The Kaplan–Meier curves illustrate the PFS for the study population in the first-line setting. The y-axis represents the *survival probability* (the proportion of patients who remained progression-free), while the x-axis represents *time in months*.

The median PFS in the first-line setting was reported to be 20.14 months (95% CI: 14.65–30.49) with a median follow-up time of 21.8 months. The probability of remaining progression-free in the first-line setting steadily decreased throughout the 60 months across all treatment groups, as demonstrated in Figure 2.

The AI group showed higher survival probability during the early treatment period, with the decline starting after approximately 15 months, and around 20–25% of patients remained progression-free at 50 months, while the SERM group exhibited PFS outcomes similar to those of the AI group during the first 30 months, with the curve showing a gradual decline. The SERD group demonstrated the shortest PFS, with a steep decline in survival probability within the first 18 months. The curve falls below 0.2 at approximately 20 months, indicating that most patients in this group experienced disease progression relatively early (Figure 2).

Compared to AIs, SERD treatment was associated with a significantly higher risk of progression (HR = 2.55; 95% CI: 1.38–4.74; *p* = 0.00299), whereas no statistically significant difference was observed between SERMs and AIs (HR = 1.11; 95% CI: 0.62–2.01; *p* = 0.7219) (Figure 2).

#### 3.2.2. Second-Line Setting

**Figure 3:**
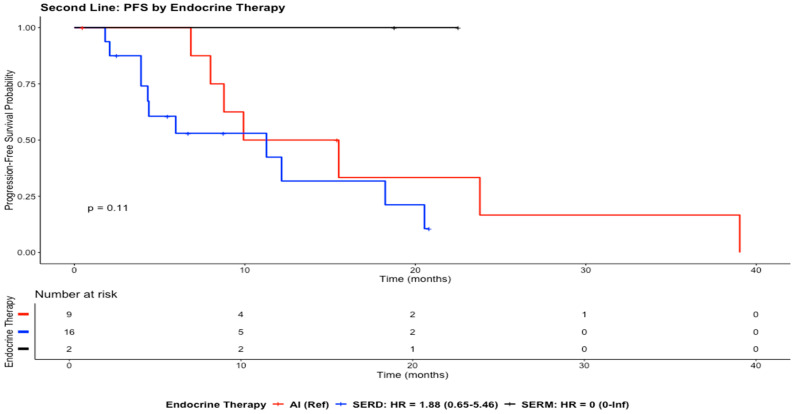
Kaplan–Meier curves illustrate PFS for the study population in the second-line setting.

The median PFS was 11.3 months (95% CI: 7.98–NA). Kaplan–Meier analysis showed a decline in progression-free survival over time across all endocrine therapy groups. The AI and SERD groups both demonstrated decreasing progression-free survival over the follow-up period, with the SERD group tending to decline earlier. The SERM group appeared to maintain a high progression-free survival probability throughout follow-up (Figure 3).

Compared with AIs, SERD treatment was associated with a higher risk of progression, although the difference was not statistically significant (HR = 1.88; 95% CI: 0.65–5.46; *p* = 0.2456). Likewise, no statistically significant difference was observed between SERMs and AIs (HR ≈ 0; 95% CI: 0.00–Inf; *p* = 0.9984) (Figure 3).

#### 3.2.3. Predictors of PFS

In the overall cohort (including both first- and second-line therapy), several factors were identified for inclusion in the Cox regression model based on the log-rank test. The analysis identified several significant predictors, including age, BMI, type of ET, and family history of cancer. Cancer type, history of venous thromboembolism (VTE), and line of therapy also showed significant associations with PFS. These findings highlight the influence of both patient characteristics and treatment-related factors on disease progression (Table 2).

A multivariable Cox proportional hazards model was applied to adjust for confounders and evaluate independent effects. This model showed that patients who received an SERD (Fulvestrant) had a higher risk of progression compared with those on AIs (HR = 2.33; 95% CI: 1.21–4.47; *p* = 0.0112). Age demonstrated a borderline association with improved PFS (HR = 0.98; *p* = 0.0522), indicating a potential trend toward better outcomes in older patients, as shown in Table 3.

### 3.3. Overall Survival

#### 3.3.1. First-Line Setting

The median OS in the first-line setting was approximately 53.1 months (95% CI: 41.2–NA) indicating that more than 50% of patients were alive at the end of follow-up.

**Figure 4:**
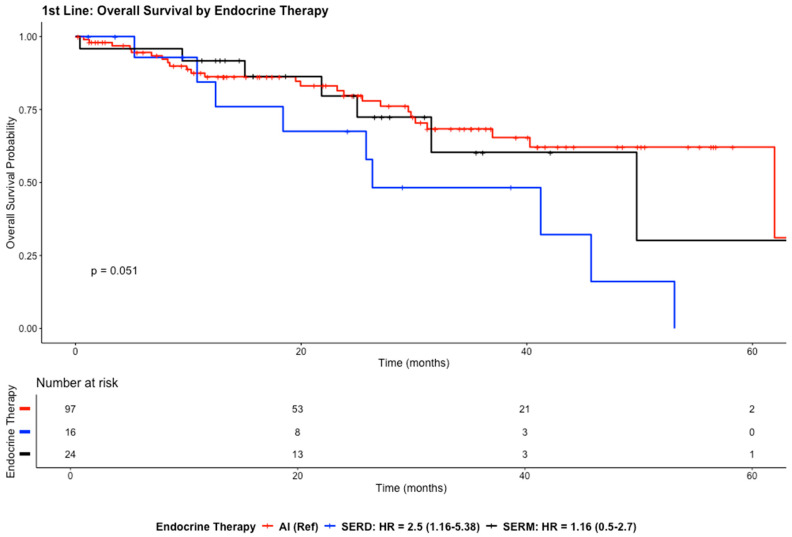
Kaplan–Meier curves illustrate OS for the study population in the first-line setting.

Compared with AIs, SERD treatment was associated with a significantly higher risk of death (HR = 2.50; 95% CI: 1.16–5.38; *p* = 0.01882), whereas no statistically significant difference was observed between SERMs and AIs (HR = 1.16; 95% CI: 0.50–2.70; *p* = 0.72671) (Figure 4).

#### 3.3.2. Second-Line Setting

In the second-line setting, the median OS was 23.7 months (95% CI: 18.5–NA). Kaplan–Meier analysis showed differences in overall survival across endocrine therapy groups during follow-up. Patients treated with AIs appeared to have the most favorable survival pattern, whereas those receiving SERDs showed a steeper decline in survival probability over time. The SERM group appeared to maintain a high survival probability throughout follow-up.

**Figure 5:**
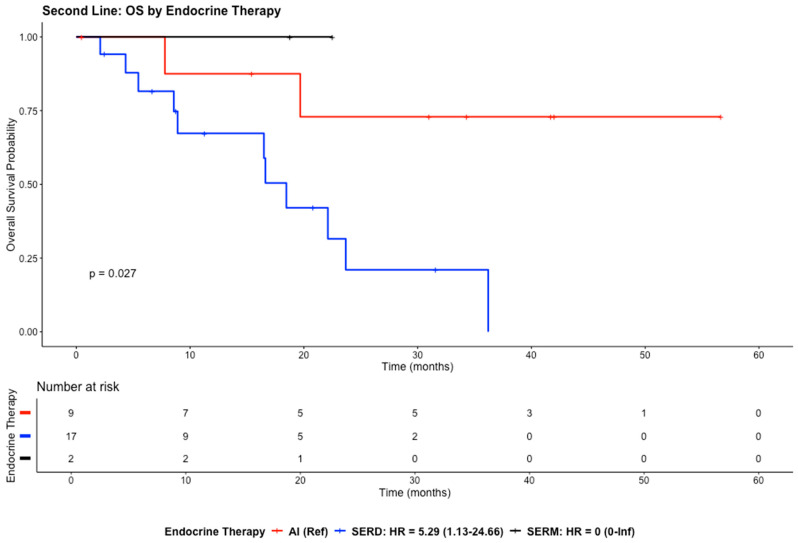
The Kaplan–Meier curves illustrate the OS for the study population in the second-line setting.

The SERD group demonstrated a significantly higher risk of death (HR = 5.29; 95% CI: 1.13–24.66; *p* = 0.034) compared with the AI group. In contrast, no statistically significant difference was observed between SERMs and AIs (HR ≈ 0; 95% CI: 0.00–Inf; *p* = 0.998) (Figure 5).

#### 3.3.3. Predictors of OS

In the overall cohort (including both first- and second-line therapy), several variables demonstrated a statistically significant association with OS. These included BMI, cancer type, type of endocrine therapy, family history of cancer, and ECOG performance status. Line of therapy, metastatic location, and BRCA mutation status were also significant predictors (Table 4).

**Figure 6:**
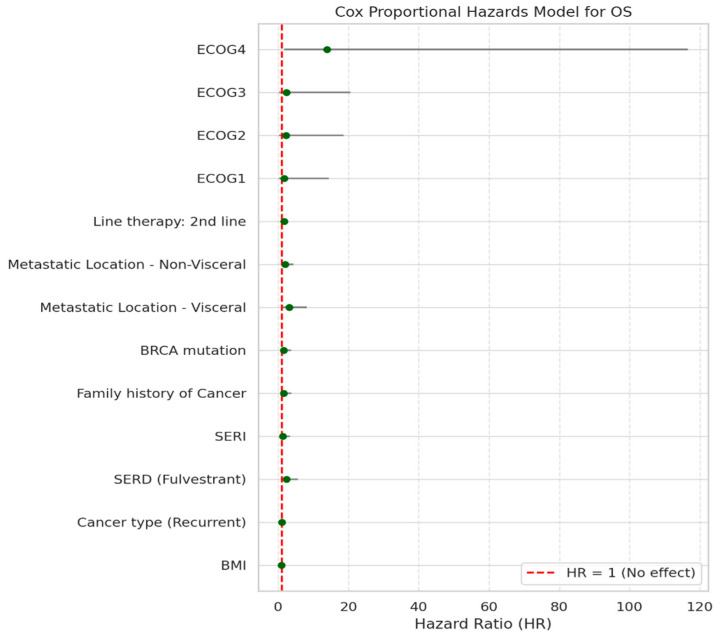
Forest plot of hazard ratios for factors associated with OS based on Cox proportional hazards analysis. The dashed vertical line indicates HR = 1 (no effect).

In the multivariate Cox regression analysis, several variables showed varying levels of association with OS (Figure 6).

**Figure 7:**
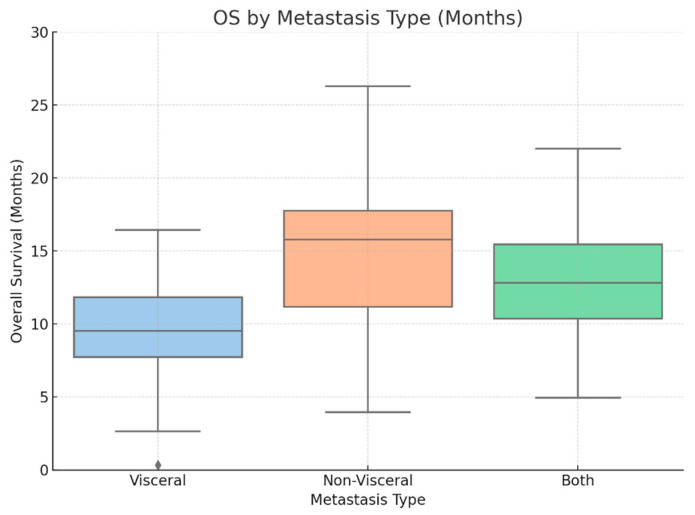
Box plot comparing OS among patients with visceral, non-visceral, and both metastasis types. Non-visceral disease was associated with longer survival.

Notably, visceral metastasis was significantly associated with worse OS (HR = 3.087, 95% CI: 1.169–8.153, *p* = 0.0229), indicating that patients with visceral metastases had over three times the risk of death compared with other patients. This finding aligns with the box plot (Figure 7).

**Figure 8:**
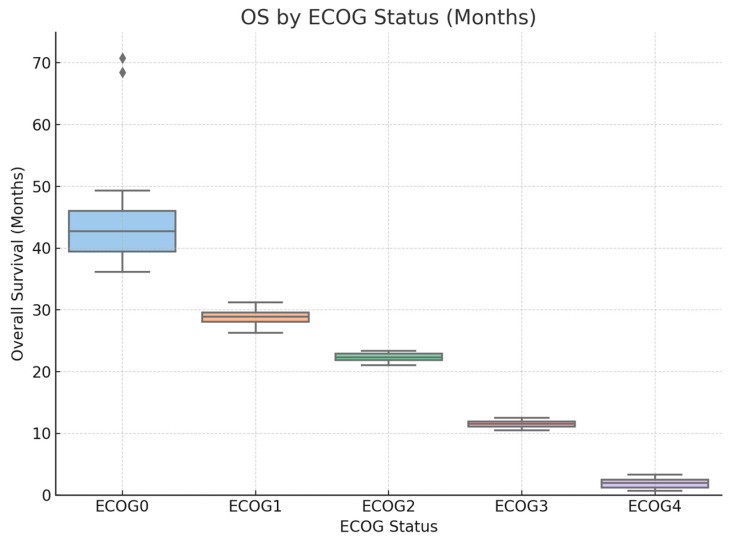
Patients with lower ECOG scores (0–1) demonstrated significantly longer OS compared with those with higher scores (≥2).

Similarly, poor functional status (ECOG 4) was strongly associated with reduced OS (HR = 13.856, 95% CI: 1.647–16.587, *p* = 0.0156), as illustrated in Figure 8.

Other variables, including BMI, cancer type, endocrine therapy, family history of cancer, BRCA mutation status, and line of therapy (Table 5), did not reach statistical significance (*p* > 0.05), although trends were observed. For instance, patients on an SERD (fulvestrant) showed a higher hazard ratio (HR = 2.302, *p* = 0.0678), and those with non-visceral metastases (HR = 2.008, *p* = 0.0696) also showed a trend toward poorer outcomes without statistical significance.

### 3.4. Toxicity Profile

Among the 169 patients included in the analysis, 52.7% experienced at least one palbociclib-related adverse event, while 47.3% had no documented toxicity. Neutropenia was the most common adverse event (45.6%), with 39.1% experiencing grade ≥ 3. Anemia was reported in 5.9% of patients. Other adverse events included leukopenia (5.3%), back pain (5.3%), joint pain (4.7%), and constipation (4.7%). Febrile neutropenia occurred in 1.2% of patients (Table 6).

Higher doses were associated with more adverse events. Most neutropenia cases occurred at 125 mg (92.2%), with fewer cases at 100 mg (5.2%) and 75 mg (1.3%). Similarly, anemia was most frequent at 125 mg (70.0%). Notably, all major adverse events, including febrile neutropenia and thromboembolic events, were observed only at the 125 mg dose.

The management strategies revealed that dose reduction (44 interventions) and holding medication (26 interventions) were the most used approaches. Permanent discontinuation was only documented in three cases.

## 4. Discussion

This retrospective analysis provides insights into the real-world effectiveness and safety of palbociclib combined with ET in females with HR+/HER2-negative MBC at a tertiary care center in Saudi Arabia. While multiple global trials have established efficacy [14,23], local evidence remains limited.

### 4.1. Progression-Free Survival

In the current study, patients who received palbociclib in the first-line setting demonstrated a longer median PFS (20.1 months) compared with those treated in the second-line setting (11.3 months). This finding is consistent with results from the PALOMA trials. PALOMA-2, which evaluated palbociclib plus letrozole as first-line therapy, reported a median PFS of 24.8 months [14], while PALOMA-3, which enrolled patients who progressed on prior endocrine therapy, reported a shorter PFS of 9.2 months with palbociclib plus fulvestrant [23]. The PFS outcomes observed in this study fall within this expected range and further support the effectiveness of palbociclib.

Regionally, a real-world study from Qatar by Al-Ziftawi et al. evaluated 108 patients with HR+/HER2− MBC treated with either CDK 4/6 inhibitors (palbociclib [75%] and ribociclib [25%]) in first-line and second-line treatment and beyond [17]. Among those receiving Palbociclib at any line of therapy, the PFS was 17.85 months. In comparison, the current study stratified outcomes by line of therapy, demonstrating numerically longer survival in the first-line setting and shorter survival in the second-line setting.

In Saudi Arabia, the study by Al-Foheidi et al. reported a median PFS of 16.3 months in 97 patients receiving palbociclib following prior endocrine therapy (second-line setting) [18]. The reported PFS is numerically higher than the one reported in this study. Notably, inclusion in Al-Foheidi et al.’s study required only at least one cycle of therapy, whereas the current study required a minimum of three cycles, potentially allowing for a more robust assessment of outcomes.

### 4.2. Overall Survival

The OS outcomes differed by line of therapy. Patients treated in the first-line setting achieved a longer median OS (53.1 months) compared with those receiving second-line therapy (23.7 months). These findings align with PALOMA-2, where median OS approached 54 months, and PALOMA-3, which reported a median OS of 34.9 months [14,23]. The shorter OS observed in the second-line group likely reflects more advanced disease, prior treatment exposure, and endocrine resistance. Furthermore, the shorter OS observed in these real-world data could be attributed to the inclusion of fragile patients (ECOG 3–4).

In Al-Ziftawi et al.’s study, the OS was 29.82 months [17]. Similar to the case in PFS, the current study stratified outcomes by line of therapy, demonstrating numerically longer survival in the first-line setting and shorter survival in the second-line setting. Furthermore, Al-Foheidi et al. reported a median OS of 19.6 months in patients who received Palbociclib in the second-line setting [18]. This is a numerically shorter survival compared with the current findings. Differences in baseline characteristics and follow-up duration may account for variations compared with the current study.

Additionally, the current study expands on regional findings by incorporating Cox regression modeling and stratified survival analysis. Visceral metastasis and poor ECOG performance status were identified as independent predictors of reduced OS. Although poor ECOG performance status was associated with a markedly increased hazard ratio, the wide confidence interval reflects the small sample size in this subgroup and warrants cautious interpretation.

This further supports the effectiveness of palbociclib in real-world practice while highlighting the influence of factors such as comorbidities, prior treatments, and baseline performance status on clinical outcomes.

The observed differences between first- and second-line outcomes highlight the importance of early initiation of CDK4/6 inhibitors, as supported by clinical trial evidence and international treatment guidelines. Patients treated earlier in the disease course are more likely to achieve prolonged benefit, whereas later-line use may be limited by disease burden and endocrine resistance.

### 4.3. Impact of Companion Endocrine Therapy on Outcomes

In the study, Fulvestrant was used in 19.5% of the patients combined with palbociclib, which appeared to be conducive to PFS and OS compared with AIs and SERMs. Patients receiving Fulvestrant had more than double the risk relative to those on other ET in the multivariable analysis. Fulvestrant was more commonly administered in later lines of therapy, where patients often have more advanced or treatment-resistant disease and less favorable prognostic features. Therefore, the observed differences in outcomes may reflect confounding. Although multivariable adjustments were performed, confounding cannot be excluded, particularly given the absence of a comprehensive stratified analysis based on treatment line.

This observation is consistent with the PALOMA-3 trial, which also reported modest benefits when palbociclib was combined with Fulvestrant in heavily pretreated populations (median PFS of 9.2 months vs. 3.8 months with placebo) [23]. In line with these findings, a Polish real-world study in the first-line setting across all CDK4/6 inhibitors (with palbociclib representing ~34%) reported substantially worse outcomes in the fulvestrant cohort compared with aromatase inhibitors (median PFS of 14.4 vs. 30.3 months) [24].

This may confound comparative analyses, as patients receiving Fulvestrant are often those with more advanced or refractory disease and poor prognostic factors. However, differences in patient populations and study design should be considered when interpreting these comparisons. Clinical trials typically enroll highly selected patients under controlled conditions, whereas real-world studies, such as the current analysis, include more heterogeneous populations with varying comorbidities, prior treatments, and treatment settings. Future studies with stratified analyses by treatment line are warranted to further clarify this association.

### 4.4. Toxicity Profile

Regarding safety, neutropenia was the most common AE and was predominantly grades 3–4 in severity, consistent with the known safety profile of palbociclib [14]. However, the incidence of neutropenia may be affected by the lack of data on granulocyte colony-stimulating factor (G-CSF) use, which was not collected in this study. Moreover, higher doses were associated with more AEs, particularly neutropenia, which was most frequent at the 125 mg dose compared with 100 mg and 75 mg. While this aligns with the PALOMA trials, which consistently report high rates of hematological toxicity [14,23], our real-world data offer reassurance regarding its manageability. Dose modifications were implemented in response to adverse events, including dose reduction and temporary treatment interruption, particularly for hematological toxicities such as neutropenia. This supports the continued use of palbociclib, even in populations with comorbidities such as diabetes (30.2%) and hypertension (49.7%), which were commonly seen in this population. Additionally, back pain was reported in three patients (1.8%) and is likely attributable to bony metastasis.

Neutropenia was reported at a higher rate in the Qatar study (56.8%) compared with the current study (45.6%), while febrile neutropenia remained uncommon (2.8% vs. 1.2%). Thrombocytopenia (3.7%) and QT prolongation (1.2%) were noted in the Qatar study, whereas anemia, leukopenia, arthralgia and constipation were more common in this study [17]. These slight variations may reflect differences in baseline performance status, comorbidities, and supportive care.

Additionally, while the previous Saudi study reported a 30% dose reduction rate without reporting detailed safety data, their study observed no discontinuations [18]. In comparison, our findings demonstrate a lower dose reduction rate (26%) and a minimal discontinuation rate (1.2%), supported by a detailed toxicity profile. These results further highlight the safety and tolerability of palbociclib in real-world clinical settings.

This study has some limitations inherent to retrospective analysis. Incomplete documentation, missing data and variability in follow-up duration may have introduced bias and affected outcome precision. Moreover, heterogeneity in treatment lines, prior therapies, and patient characteristics complicate direct comparisons with randomized trials or other regional reports. Although the objective response rate (ORR) was initially included as a secondary objective in the study proposal, it was excluded from the final analysis due to a high percentage of missing or incomplete data in patient electronic health records (EHRs), which limited the reliability of objective response assessment.

Despite these limitations, the present study provides several important incremental contributions to the existing literature. First, our study included a larger patient cohort (169 patients compared with 97 patients in the earlier study). Second, the longer follow-up duration in the current analysis enables a more robust assessment of survival outcomes. Most importantly, the use of multivariate statistical analysis, and Kaplan–Meier survival analysis allowed for reliable estimation of progression-free and overall survival, while Cox regression modeling enabled identification of new independent prognostic factors influencing outcomes. Together, these analytical methods strengthen the validity of the findings compared with the previous Saudi data [18].

Recent studies have explored the use of artificial intelligence and machine learning approaches to improve prognostic assessment in breast cancer, including models based on histopathological images for relapse prediction [25,26]. While these approaches show promising predictive performance, they primarily rely on imaging data. In contrast, our study identifies clinically relevant independent prognostic factors derived from real-world data, such as treatment patterns and patient characteristics. These variables may serve as valuable complementary inputs for future machine learning models, particularly in improving treatment selection and outcome prediction in routine clinical practice.

Overall, this study addresses key limitations of previous local studies and offers clinically relevant data to support treatment decision making. Notably, while our findings are broadly consistent with global evidence, they also highlight region-specific differences in treatment patterns and outcomes in Saudi Arabia.

## 5. Conclusions

Palbociclib in combination with ET remains an effective treatment option for HR+/HER2− MBC. Survival outcomes differed by line of therapy, with improved PFS and OS observed in patients treated in the first-line setting compared with second-line therapy. Visceral metastasis and high ECOG status were associated with poor survival outcomes, which may guide future treatment decisions. The toxicity profile was consistent with known safety data, with neutropenia being the most frequent AE. These findings contribute valuable regional data to the growing evidence base and support the use of palbociclib.

## Figures and Tables

**Table 1:** Baseline patient demographics and disease history.

Variable	Mean +/− SD	Median	Min, Max
**Age**	58.36 +/− 10.93351 years	58.0 year	35, 91 year
**Body Mass Index (BMI)**	30.24 +/− 7.893218 kg/m^2^	29.60 kg/m^2^	15.18, 58.27 kg/m^2^
**Variable**	**Frequency (%)**		
**ECOG Performance Status**			
0	9 (5.32%)		
1	55 (32.54%)		
2	52 (30.77%)		
3	18 (10.65%)		
4	16 (9.47%)		
Not Recorded	19 (11.24%)		
**Hypertension**			
Yes	84 (49.7%)		
No	85 (50.3%)		
**Hyperlipidemia**			
Yes	38 (22.5%)		
No	131 (77.5%)		
**Diabetes**			
Yes	77 (45.6%)		
No	92 (54.4%)		
**Atrial Fibrillation**			
Yes	5 (3.0%)		
No	164 (97.0%)		
**History of VTE**			
Yes	12 (7.1%)		
No	157 (92.9%)		
**Chronic Kidney Disease**			
Yes	10 (5.9%)		
No	159 (94.1%)		
**Liver Dysfunction**			
Yes	4 (2.4%)		
No	165 (97.6%)		
**History of Cancer**			
Yes	22 (13.0%)		
No	120 (71.0%)		
Not Mentioned	27 (16.0%)		
**Family History of Cancer**			
Yes	26 (15.4%)		
No	98 (58.0%)		
Not Mentioned	45 (26.6%)		
**BRCA Mutation**			
Yes	38 (22.5%)		
No	73 (43.2%)		
Not Mentioned	58 (34.3%)		
**Cancer Type**			
Primary	121 (71.6%)		
Recurrent	48 (28.4%)		
**Line Therapy**			
First Line	139 (82.2%)		
Second Line	30 (17.8%)		
**Menopausal status**			
**–** **Premenopausal**	29 (17.2%)		
**–** **Perimenopausal**	8 (4.7%)		
**–** **Postmenopausal**	131 (77.5%)		
**–** **Hysterectomy**	1 (0.6%)		
**Metastatic location**			
**–** **Visceral Metastases**	25 (14.8%)		
**–** **Non-Visceral Metastases**	65 (38.5%)		
**–** **Both**	79 (46.7%)		
**Endocrine therapy**			
**–** **AIs**	110 (65.1%)		
**–** **SERDs**	33 (19.5%)		
**–** **SERMs**	26 (15.4%)		

**Table 2:** Log-rank test for PFS.

Variable	*p*-Value
Age	<0.0000 *
BMI	0.0000 *
Cancer type (primary vs. recurrent)	0.0109 *
Type of endocrine therapy	0.0001 *
Family history of cancer	0.0019 *
History of VTE	0.0337 *
Line therapy (1st vs. 2nd line)	0.0129 *
Hypertension	0.7598
Hyperlipidemia	0.4092
Diabetes	0.2106
Chronic kidney disease (CKD)	0.5452
ECOG performance status	0.7104
Menopausal status	0.6950
Metastatic locations	0.1125
BRCA mutation	0.8085

* Statistically significant at *p* < 0.05 (log-rank test for PFS).

**Table 3:** Cox proportional model for PFS.

Variable	HR	(95% CI)	*p*-Value
Age	0.98	(0.96–1.00)	0.0522
BMI	0.98	(0.96–1.01)	0.2524
Cancer type (recurrent)	0.88	(0.48–1.61)	0.6870
Endocrine therapy: selective estrogen receptor degrader (Fulvestrant)	2.33	(1.21–4.47)	0.0112 *
Endocrine therapy: selective estrogen receptor modulator (Tamoxifen)	0.83	(0.44–1.57)	0.5744
Family history of cancer	0.61	(0.29–1.27)	0.1868
History of VTE: yes (reference: no VTE)	1.62	(0.83–3.18)	0.1606
Line therapy: 2nd line (reference: 1st line)	1.14	(0.63–2.05)	0.6629

* Statistically significant at *p* < 0.05.

**Table 4:** Log-rank test for OS.

Variable	*p*-Value
BMI	<0.0000 *
Cancer type	0.0090 *
Endocrine therapy	0.00003 *
Family history of cancer	0.0016 *
ECOG performance status	0.00023 *
Line therapy	0.0177 *
Metastatic locations	0.00822 *
BRCA mutation	0.0148 *
Age	0.1151
Hypertension	0.3070
Hyperlipidemia	0.0671
Diabetes	0.2571
CKD	0.8910
Menopausal status	0.4367
History of VTE	0.6830

* Statistically significant at *p* < 0.05 (log-rank test for OS).

**Table 5:** Cox proportional model for OS.

Variable	Hazard Ratio	95% Confidence Interval	*p*-Value
BMI	0.970	(0.934–1.007)	0.1129
Cancer type (recurrent)	1.028	(0.9724–1.028)	0.9468
Endocrine therapy:			
– SERD (Fulvestrant)	2.302	(0.941–5.632)	0.0678
– SERM (Tamoxifen)	1.305	(0.506–3.364)	0.5818
Family history of cancer	1.529	(0.615–3.798)	0.3606
BRCA mutation	1.545	(0.645–3.699)	0.3287
Metastatic location—visceral	3.087	(1.169–8.153)	0.0229 *
Metastatic location—non-visceral	2.008	(0.946–4.263)	0.0696
Line therapy: 2nd line (reference: 1st line)	1.766	(0.569–1.766)	0.1419
ECOG1	1.715	(0.205–14.379)	0.6191
ECOG2	2.188	(0.258–18.545)	0.4728
ECOG3	2.4164	(0.283–20.606)	0.4198
ECOG4	13.856	(1.647–116.587)	0.0156 *

* Statistically significant at *p* < 0.05.

**Table 6:** Palbociclib-related toxicity.

Toxicity	Total Patients (Any Grade)	Grades 1–2 (%)	Grades ≥ 3 (%)	% of Total Population (n = 169)
**Neutropenia**	77	11 (14.3%)	66 (85.7%)	45.6%
**Anemia**	10	7 (70.0%)	3 (30.0%)	5.9%
**Leukopenia**	9	5 (55.6%)	4 (44.4%)	5.3%
**Back Pain**	9	7 (77.8%)	2 (22.2%)	5.3%
**Joint Pain (Arthralgia)**	8	5 (62.5%)	3 (37.5%)	4.7%
**Constipation**	8	8 (100.0%)	0 (0.0%)	4.7%
**Fatigue**	8	8 (100.0%)	0 (0.0%)	4.7%
**Thrombocytopenia**	5	1 (20.0%)	4 (80.0%)	3.0%
**Nausea**	3	3 (100.0%)	0 (0.0%)	1.8%
**Diarrhea**	3	3 (100.0%)	0 (0.0%)	1.8%
**Thromboembolic Events**	2	2 (100.0%)	0 (0.0%)	1.2%
**Febrile Neutropenia**	2	0 (0.0%)	2 (100.0%)	1.2%
**Pancytopenia**	1	0 (0.0%)	1 (100.0%)	0.6%
**Decreased Appetite**	1	1 (100.0%)	0 (0.0%)	0.6%
**Dyspnea**	1	1 (100.0%)	0 (0.0%)	0.6%
**Skin Rash**	1	0 (0.0%)	1 (100.0%)	0.6%

## Data Availability

The datasets generated and analyzed during the current study are not publicly available due to patient confidentiality restrictions but are available from the corresponding author upon reasonable request and with appropriate institutional approval.
